# Supplementary material for: Crucial Parameters for Immunopeptidome Characterization: A Systematic Evaluation
Source: Int J Mol Sci. 2024 Sep 3;25(17):9564. doi: 10.3390/ijms25179564 (PMC11395153; doi:10.3390/ijms25179564)
Supplement: Supplementary file 1 [file ijms-25-09564-s001.zip › IJMS_Immunopeptidomics_Sup_Info_vs1.pdf]

International Journal of *Molecular Sciences*

MDPI

Article

Crucial Parameters for Immunopeptidome Characterization: A systematic evaluation

Supporting Information

Pablo Juanes-Velasco[1], Carlota Arias-Hidalgo[1], Marina L. García-Vaquero[1], Janet Sotolongo-Ravelo[2], Teresa Paíno[2,3], Quentin Lecrevisse[1], Alicia Landeira-Viñuela[1], Rafael Góngora[1], Ángela-Patricia Hernández[1,4], Manuel Fuentes[1,5]*

[1] Department of Medicine and General Cytometry Service-Nucleus, CIBERONC, Cancer Research Centre (IBMCC, CSIC/USAL) Consejo Superior de Investigaciones Cientificas (CSIC) Universidad de Salamanca (USAL), and Instituto de Investigacion Biomedica de Salamanca (IBSAL), 37007 Salamanca, Spain
[2] Oncohematology Group, Cancer Research Center (IBMCC/CSIC/USAL/IBSAL), 37007 Salamanca, Spain.
[3] Department of Physiology and Pharmacology, University of Salamanca, 37007 Salamanca, Spain.
[4] Department of Pharmaceutical Sciences: Organic Chemistry, Faculty of Pharmacy, University of Salamanca, CIETUS, IBSAL, Salamanca, Spain.
[5] Proteomics Unit, Cancer Research Centre (IBMCC/CSIC/USAL/IBSAL), 37007 Salamanca, Spain.

* Correspondence: mfuentes@usal.es

Keywords: Immunopeptidomics; Immunopeptidome; HLA; MHC; LC-MS/MS.

Citation: To be added by editorial staff during production.

Academic Editor: Firstname Lastname

Received: date
Revised: date
Accepted: date
Published: date

Copyright: © 2024 by the authors. Submitted for possible open access publication under the terms and conditions of the Creative Commons Attribution (CC BY) license (https://creativecommons.org/licenses/by/4.0/).

1. Supplementary Data

In addition to the materials and methods that have already been discussed in the main text, other reagents and equipment that have been used are listed below.

1.1. Materials.

Sigma-Aldrich, Missouri, USA: Tetramethylethylenediamine (TEMED); Bovine Serum Albumin (BSA); Sodium Dodecylsulfate (SDS); Tween-20, Methanol.

ThermoFisher Scientific, Massachusetts, USA: PageRulerTM Plus Prestained Protein Marker; SuperSignalTM West Pico PLUS Chemiluminescent Substrate; Glycine; Acetonitrile, LC/MS Grade; epTIPS LoRetention Tips, 1000 µL/Eppendorf; epTIPS LoRetention Tips, 200 µL/Eppendorf; Tube LoBind 2 mL/Eppendorf; Tube LoBind 1.5 mL/Eppendorf; Water, LC/MS Grade.

Bio-Rad Laboratories, Inc, California, USA: Trans-Blot Turbo Transfer System; Mini-PROTEAN® Tetra Vertical Electrophoresis Cell; Mini-PROTEAN® Tetra Cell Casting Module; PowerPacTM Basic Power Supply.

National Diagnostics, Atlanta, USA: ProtoGel (30%).

Roche Diagnostics, Risch-Rotkreuz, Switzerland: 1,4-Dithiothreitol (DTT); Tris Base (C4H11NO3).

Merck, Darmstadt, Germany: Dimethyl sulfoxide (DMSO); Absolute ethanol.

Merk Millipore, Darmstadt, Germany: Immobilon®-FL PVDF Transfer Membrane; PureProteomeTM Magnetic Stand.

Fujifilm Corporation, Tokyo, Japan: Super RX-N Photographic Films; BAS Cassette2-2340.

BRAND GMBH + CO KG, Wertheim, Germany: Neubaer Counting Chamber without clamps.

Cancer Research Center Centralized Services (CIC- IBMCC, Salamanca, Spain): 1X Na+/K+ Phosphate Buffer Saline (PBS); 20X Tris Buffer Saline (TBS); milli-Q water; 1X Transfer Buffer (Tris-glycine, Methanol 20%); 1X Running Buffer (Tris Base0. 25M, 2M Glycine, SDS 10%), Tris/HCl Buffer pH=6.8; Tris/HCl Buffer pH=8.8; SDS Loading Buffer 5X; Coomassie Blue Solution (Coomassie Blue R-250 1g/L, Acetic Acid 10%, Methanol 40%); Coomassie Destaining Solution (Acetic Acid 10%, Methanol 40%). In addition to the consumables of the laboratory 11 of the Cancer Research Center (CIC-IBMCC, Spain).

1.2. Methods.

1.2.1. Antibody Coupling to Immunoaffinity matrix for IP

1.2.1.1. Immunoaffinity matrix #1

The immunoaffinity matrix #1 was magnetic microspheres QuantumPlex M SP Carboxyl (Bangs Laboratories Inc, Ref: 251A, USA) with high density carboxylic functional groups on the surface to covalently conjugate antibodies through their primary amines via a Luminex EDC/NHS protocol as described below [1-4].

1. Resuspend the stock uncoupled microsphere suspension according to the instructions described in the Product Information Sheet provided with your microspheres.

2. Transfer 5.0 x 10^6 of the stock microspheres to a recommended microcentrifuge tube.

3. Place the tube into a magnetic separator and allow separation to occur for 30 to 60 seconds.

4. With the tube still positioned in the magnetic separator, remove the supernatant. Take care not to disturb the microspheres.

5. Remove the tube from the magnetic separator and resuspend the microspheres in 100 µL dH2O by vortex and sonication for approximately 20 seconds.

6. Place the tube into a magnetic separator and allow separation to occur for 30 to 60 seconds.

7. With the tube still positioned in the magnetic separator, remove the supernatant. Take care not to disturb the microspheres.

8. Remove the tube from the magnetic separator and resuspend the washed microspheres in 80 µL 100 mM Monobasic Sodium Phosphate, pH 6.2 by vortex and sonication for approximately 20 seconds.

9. Add 10 µL of 50 mg/mL Sulfo-NHS (diluted in dH20) to the microspheres and mix gently by vortex.

10. Add 10 µL of 50 mg/mL EDC (diluted in dH20) to the microspheres and mix gently by vortex.

11. Incubate for 20 minutes at room temperature with gentle mixing by vortex at 10 minutes intervals.

12. Place the tube into a magnetic separator and allow separation to occur for 30 to 60 seconds.

13. With the tube still positioned in the magnetic separator, remove the supernatant. Take care not to disturb the microspheres.

14. Remove the tube from the magnetic separator and resuspend the microspheres in 250 µL of 50 mM MES, pH 5.0 by vortex and sonication for approximately 20 seconds.

15. Repeat steps 13 and 14 for a total of two washes with 50 mM MES, pH 5.0.

16. Remove the tube from the magnetic separator and resuspend the activated and washed microspheres in 100 µL of 50 mM MES, pH 5.0 by vortex and sonication for approximately 20 seconds.

17. Add 1 mg of antibody to t1 mL of the resuspended microspheres.

18. Bring total volume to 500 µL with 50 mM MES, pH 5.0.

19. Mix coupling reaction by vortex.

20. Incubate for 2 hours with mixing (by rotation) at room temperature.

21. Place the tube into a magnetic separator and allow separation to occur for 30 to 60 seconds.

22. With the tube still positioned in the magnetic separator, remove the supernatant. Take care not to disturb the microspheres.

23. Remove the tube from the magnetic separator and resuspend the coupled microspheres in 500 µL of PBS-TBN by vortex and sonication for approximately 20 seconds.

24. Optional – Incubate for 30 minutes with mixing (by rotation) at room temperature. (Note: Perform this step when using the microspheres the same day.)

25. Place the tube into a magnetic separator and allow separation to occur for 30 to 60 seconds.

26. With the tube still positioned in the magnetic separator, remove the supernatant. Take care not to disturb the microspheres.

27. Remove the tube from the magnetic separator and resuspend the microspheres in 1 mL of PBS-TBN by vortex and sonication for approximately 20 seconds.

28. Repeat steps 25. and 26. This is a total of two washes with 1 mL PBS-TBN.

29. Remove the tube from the magnetic separator and resuspend the coupled and washed microspheres in 250-1000 µL of PBS-TBN.

30. Count the number of microspheres recovered after the coupling reaction using a cell counter or hemacytometer.

31. Store coupled microspheres refrigerated at 2-8°C in the dark.

Confirmation and validation of antibody binding to the sphere was performed by flow cytometry (FACS Aria) (Supplementary Figure 1A).

1.2.1.2. Immunoaffinity matrix #2

The immunoaffinity matrix #2 was sepharose spheres (CNBr-activated Sepharose® 4B - Cytiva 17-0430-01) capable of binding antibodies by the cyanogen bromide method.

The binding of the antibody to the sepharose sphere is carried out in three steps [5]. 1. Activation of the CNBr sepharose spheres. 2. Antibody binding. 3.- Blocking and washing of the spheres bound to the antibody.

1.- Activation of the CNBr sepharose spheres.

To perform the activation of the spheres, 80 mg of them are weighed (for each sample) and transferred to a 15 mL conical tube where they will be resuspended with 5 mL of 1 mM HCl and later, with 8.5 mL more of 1 mM HCl. To maintain in rotation with this solution for 30 minutes at room temperature and at the end centrifuge at 200 xg for 2 minutes to eliminate the supernatant.

Finally, 500 µL of coupling buffer (0.5 M NaCl, 0.1M NaHCO3, pH 8.3.) is added over the pellet of beads.

2.- Antibody binding.

The spheres are transferred to a new tube and 2 mg of antibody is added. The volume is adjusted to 1000 µL with the coupling buffer. This tube is kept in rotation for 120 minutes at room temperature. At the end, it is centrifuged at 200 xg for 2 minutes to eliminate the supernatant.

3.- Blocking and washing of the spheres bound to the antibody.

Finally, 1 mL of 0.2 M glycine is added and left in rotation for 60 minutes at room temperature. When finished, centrifuge at 200 xg for 2 minutes and remove the supernatant.

1 mL of PBS is added and centrifuged at 200 xg for 2 minutes and the supernatant is removed. Finally, 1 mL of PBS is added and the spheres are kept at 4 °C until use.Confirmation and validation of antibody binding to the sphere was performed by Coomasie staining and western blotting (Supplementary Figure 1B and 1C).

Antibody binding to immunoaffinity matrices was done the same week as the assay was performed.

2. Supplementary Figures and Tables

2.1. Supplementary Figures.

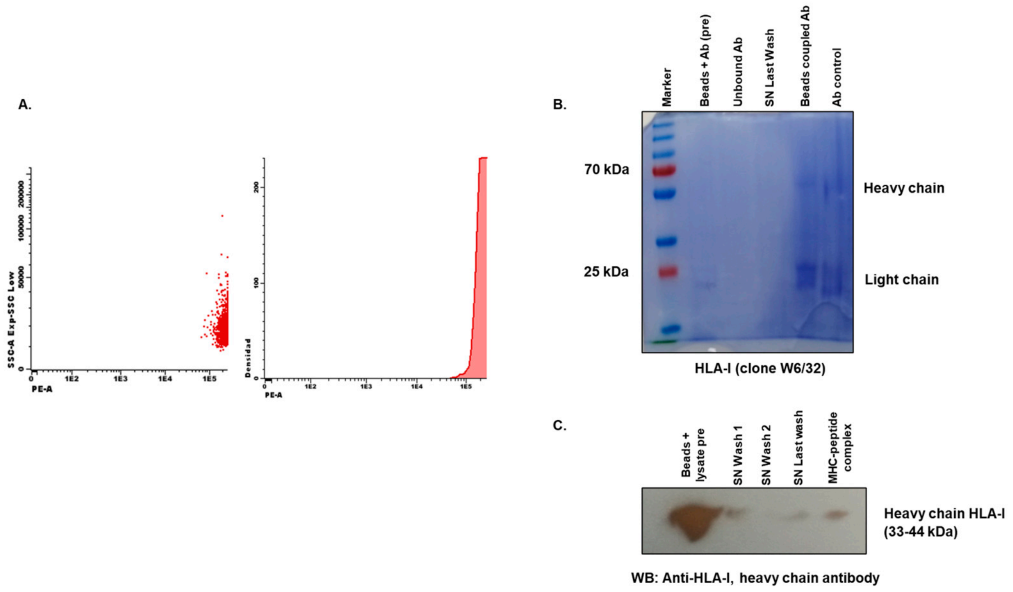

Supplementary Figure S1. Track of the antibody coupling to solid support for IP. (A). Confirmation and validation of antibody pan-HLA-I (clone W6/32) binding to QuantumPlex M SP Carboxyl magnetic spheres performed by flow cytometry (FACS Aria). (B). Coomasie 12 % SDS-PAGE gel staining to confirm and validate the antibody pan-HLA-I (clone W6/32) binding to CNBr-activated Sepharose® 4B beads. (C). Western blotting (12 % SDS-PAGE) PVDF membrane to detect signal of MHC-complexes using anti-HLA-I, heavy chain antibody (Abcam, #ab 70328, 1:5000).

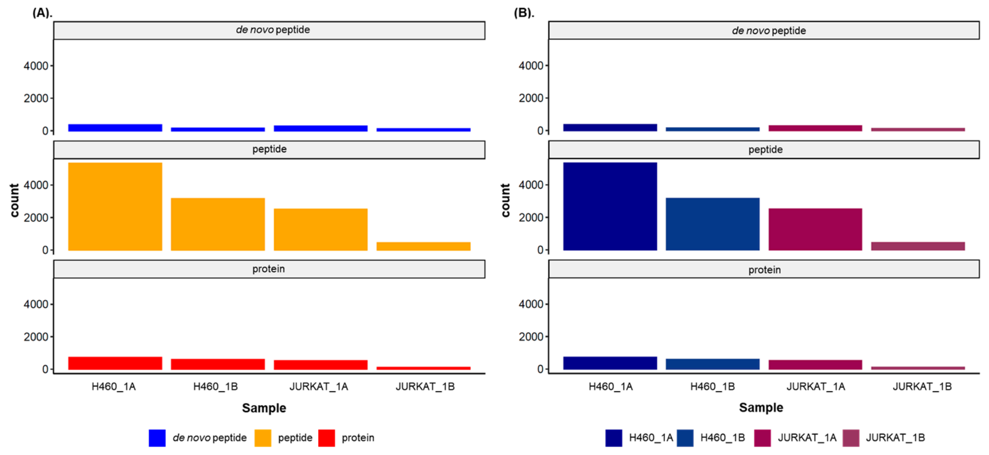

Supplementary Figure S2. Bar plots of the number of identifications in immunoaffinity matrix #1 at three levels: *de novo* peptides, peptides and proteins. (A). Count of identifications in Protocol #1 in each sample of the Immunopeptidomic assay divided by the *de novo* peptides, peptides and proteins. (B). Same information comparing the three levels in each sample of the study.

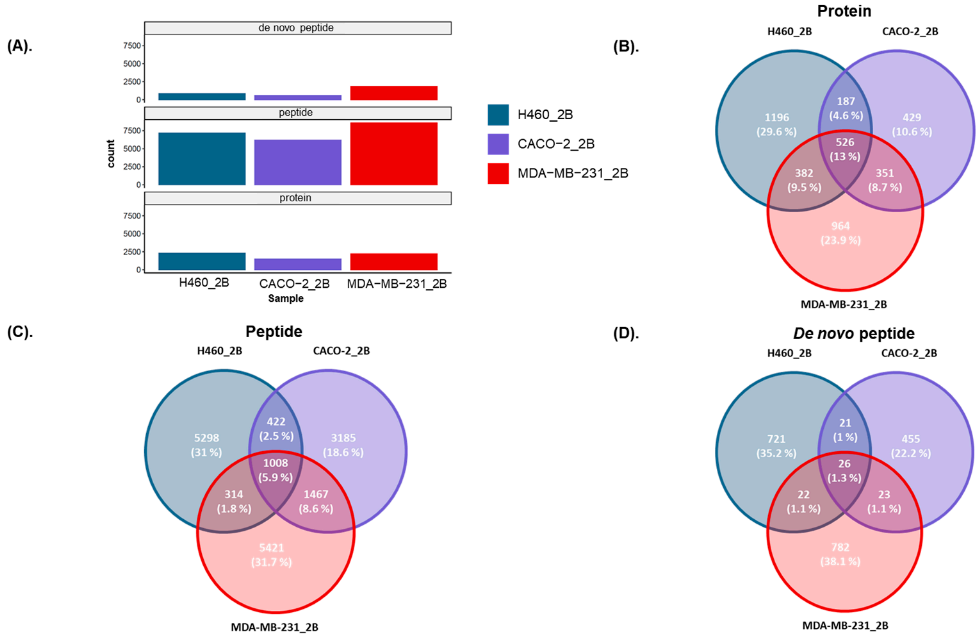

Supplementary Figure S3. Comparison between different cell lines, using the same protein extraction protocol (Protocol B) and the same immunoaffinity matrix #2). (A). Identification count of *de novo* peptides, peptides and proteins between sample H460_2B, CACO-2_2B and MDA-MB-231_2B by bar plots. (B). Proteins identified in each sample and the percentage of common proteins between H460_2B, CACO-2_2B and MDA-MB-231_2B by Venn diagrams. (C). Peptides identified in each sample and the percentage of common peptides between H460_2B, CACO-2_2B and MDA-MB-231_2B by Venn diagrams. (D). *De novo* peptides identified in each sample and the percentage of common de novo peptides between H460_2B, CACO-2_2B and MDA-MB-231_2B by Venn diagrams.

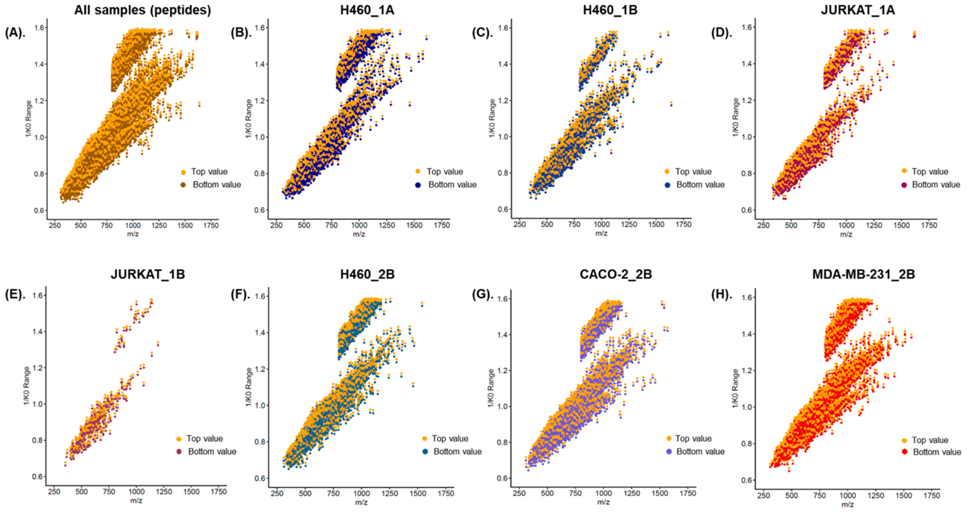

Supplementary Figure S4. Mobilograms plots of the ion mobility of the peptides versus their mass-to-charge ratio. (A). Mobilogram plots of the peptides from all the samples included in the study. (B). Mobilogram of the peptides of H460_1A. (C). Mobilogram of the peptides of H460_1B. (D). Mobilogram of the peptides of JURKAT_1A. (E). Mobilogram of the peptides of JURKAT_1B. (F). Mobilogram of the peptides of H460_2B. (G). Mobilogram of the peptides of CACO-2_2B. (H). Mobilogram of the peptides of MDA-MB-231_2B.

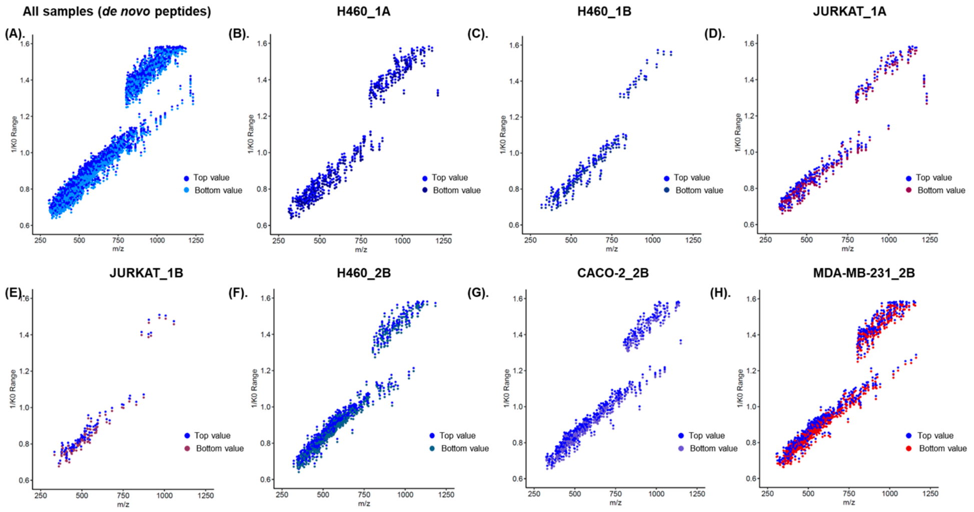

Supplementary Figure S5. Mobilograms plots of the ion mobility of the *de novo* peptides versus their mass-to-charge ratio. (A). Mobilogram plots of the *de novo* peptides from all the samples included in the study. (B). Mobilogram of the *de novo* peptides of H460_1A. (C). Mobilogram of the *de novo* peptides of H460_1B. (D). Mobilogram of the *de novo* peptides of JURKAT_1A. (E). Mobilogram of the *de novo* peptides of JURKAT_1B. (F). Mobilogram of the *de novo* peptides of H460_2B. (G). Mobilogram of the *de novo* peptides of CACO-2_2B. (H). Mobilogram of the *de novo* peptides of MDA-MB-231_2B.

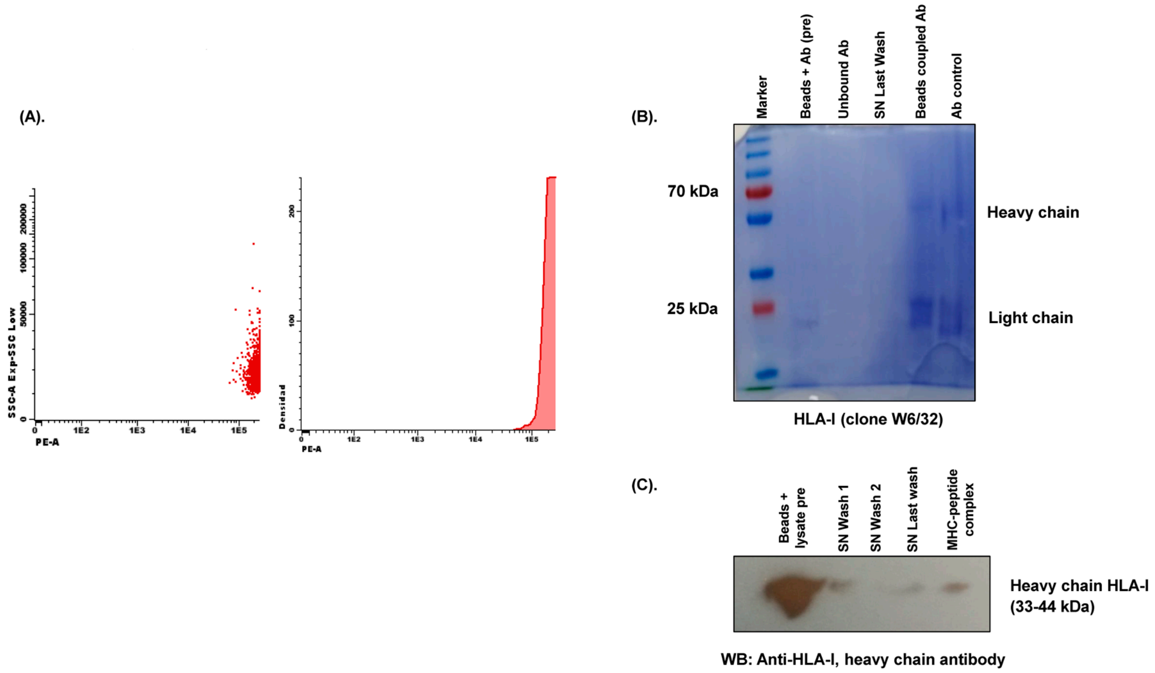

Supplementary Figure S6. Chromatograms of the samples studied. It shows the chromatograms of the different samples analyzed, where the intensity of the different analytes separated in the acquisition time can be appreciated.

2.2. Supplementary Tables.

Supplementary Table S1. Description of the proteins, peptides and *de novo* peptides identified in the tumour cell lines used in the study (FDR = 5 %). Raw information of the proteins, peptides and de novo peptides from all the samples included in the study, obtained from PEAKS Studio X Pro 10.6.

Supplementary Table S2. Information of proteins, peptides and *de novo* peptides from comparatives established. Information from each of the sample comparisons performed in the study. Results obtained from Venny 2.1.

Supplementary Table S3. Peptides length distribution between samples. Information of peptides length distribution from each of the sample of the study. It also includes the number of peptides with a length between 8 and 12 amino acids.

Supplementary Table S4. *De novo* peptides length distribution between samples. Information of *de novo* peptides length distribution from each of the sample of the study. It also includes the number of peptides with a length between 8 and 12 amino acids.

Supplementary Table S5. Peptides charge distribution between samples. Information of peptides charge distribution from each of the sample of the study. It also includes the number of peptides with a length between 8 and 12 amino acids.

Supplementary Table S6. *De novo* peptides charge distribution between samples. Information of de novo peptides charge distribution from each of the sample of the study. It also includes the number of peptides with a length between 8 and 12 amino acids.

Supplementary Table S7. Results of contaminants by CRAPome. List of proteins obtained from each study sample when analyzed by Contaminant Repository for Affinity Purification Mass Spectrometry Data (CRAPome) (http://www.crapome.org/).

Supplementary Table S8. Immunopeptidome Score selection of peptides and *de novo* peptides identified in each cell line (FDR = 5 %). Information of peptides and de novo peptides selected by the Immunopeptidome Score with a score of 3 which means best properties to be potential therapeutic targets in each cell line.

Supplementary Table S9. Immunopeptidome Score selection of peptides and *de novo* peptides identified in each cell line (FDR of 5 % and 1 %). Information of peptides and de novo peptides selected by the Immunopeptidome Score with a score of 3 which means best properties to be potential therapeutic targets in each cell line.

Supplementary Table S10. Immunopeptidome Score selection of peptides and *de novo* peptides identified in evaluation cell line (FDR = 1 %). Information of peptides and de novo peptides selected by the Immunopeptidome Score with a score of 3 which means best properties to be potential therapeutic targets in the cell line.

References

1. Fischer, M.J.E. Amine Coupling Through EDC/NHS: A Practical Approach. In *Surface Plasmon Resonance: Methods and Protocols*, DeMol, N.J., Fischer, M.J.E., Eds.; Methods in Molecular Biology; 2010; Volume 627, pp. 55-73.
2. de Jager, W.; te Velthuis, H.; Prakken, B.J.; Kuis, W.; Rijkers, G.T. Simultaneous detection of 15 human cytokines in a single sample of stimulated peripheral blood mononuclear cells. *Clinical and Diagnostic Laboratory Immunology* **2003**, *10*, 133-139, doi:10.1128/cdli.10.1.133-139.2003.
3. Giavedoni, L.D. Simultaneous detection of multiple cytokines and chemokines from nonhuman primates using luminex technology. *Journal of Immunological Methods* **2005**, *301*, 89-101, doi:10.1016/j.jim.2005.03.015.
4. Lawson, S.; Lunney, J.; Zuckermann, F.; Osorio, F.; Nelson, E.; Welbon, C.; Clement, T.; Fang, Y.; Wong, S.; Kulas, K.; et al. Development of an 8-plex Luminex assay to detect swine cytokines for vaccine development: Assessment of immunity after porcine reproductive and respiratory syndrome virus (PRRSV) vaccination. *Vaccine* **2010**, *28*, 5356-5364, doi:10.1016/j.vaccine.2010.05.016.
5. Sirois, I.; Isabelle, M.; Duquette, J.D.; Saab, F.; Caron, E. Immunopeptidomics: Isolation of Mouse and Human MHC Class I- and II-Associated Peptides for Mass Spectrometry Analysis. *Jove-Journal of Visualized Experiments* **2021**, doi:10.3791/63052.
