# Supplementary figures and images for: Crucial Parameters for Immunopeptidome Characterization: A Systematic Evaluation
Source: Int J Mol Sci. 2024 Sep 3;25(17):9564. doi: 10.3390/ijms25179564 (PMC11395153; doi:10.3390/ijms25179564)

Supplementary Figure S1

(A).

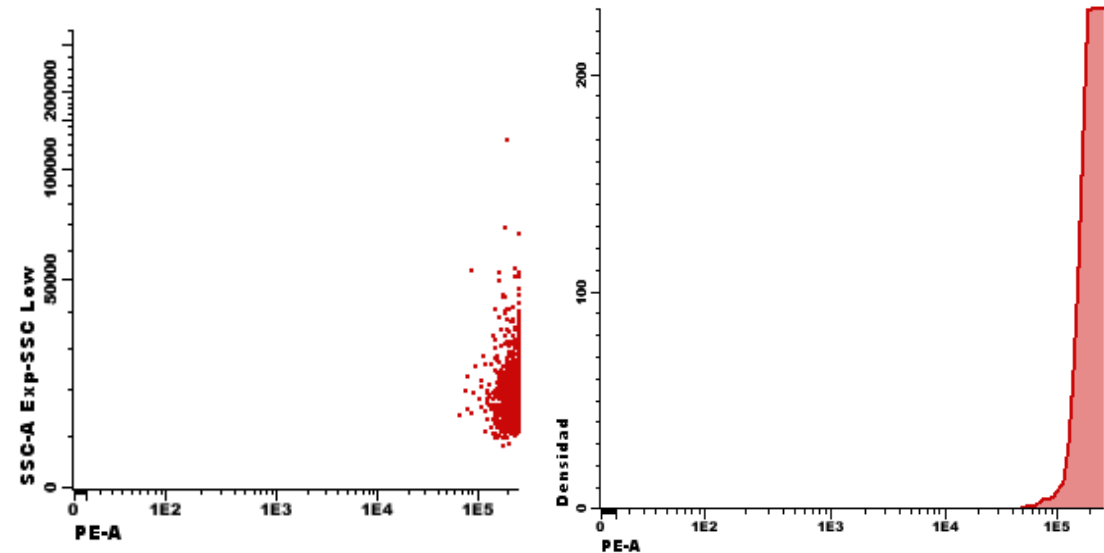

(B).

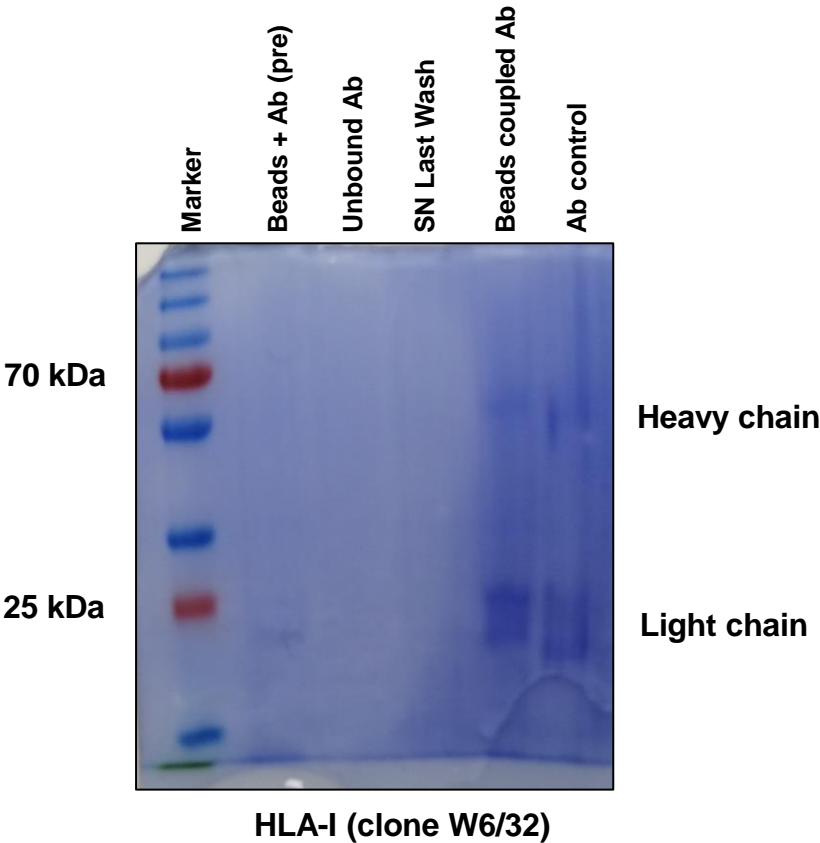

(C).

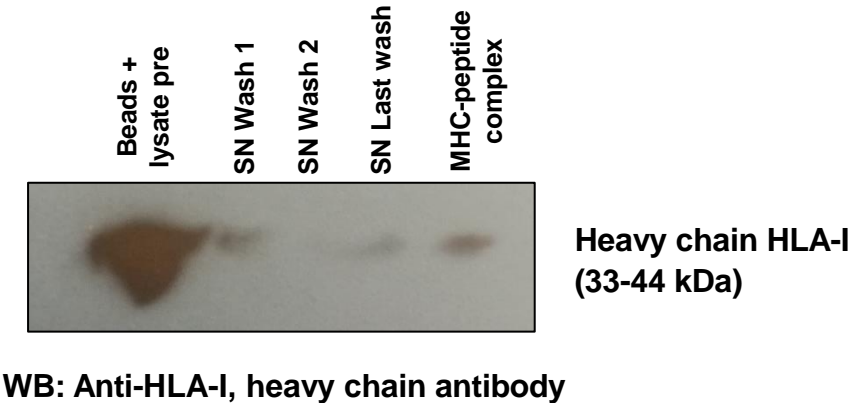

Supplement: Supplementary file 1 [file ijms-25-09564-s001.zip › Supplementary Figure S1.pdf]

Supplementary Figure S2

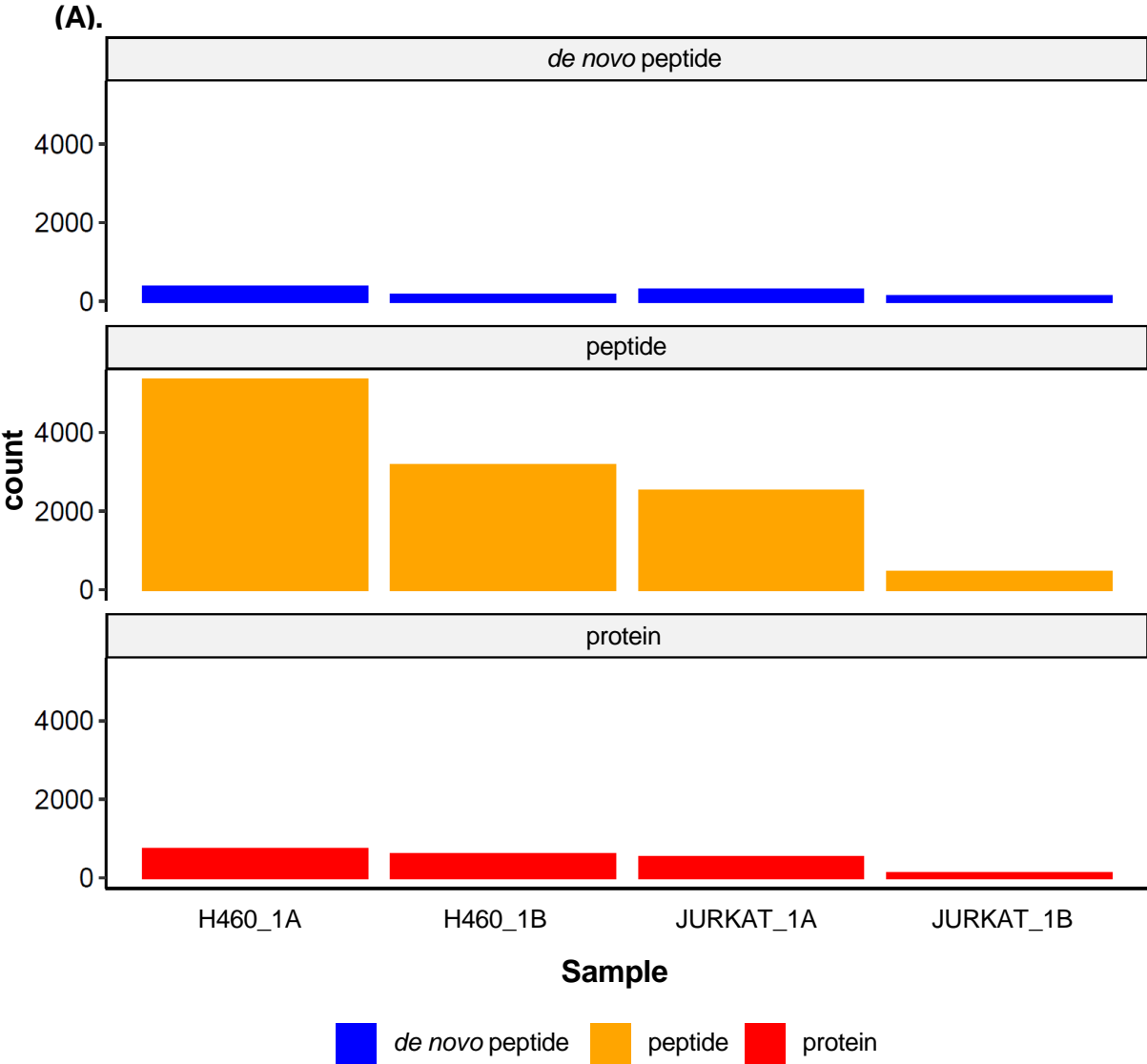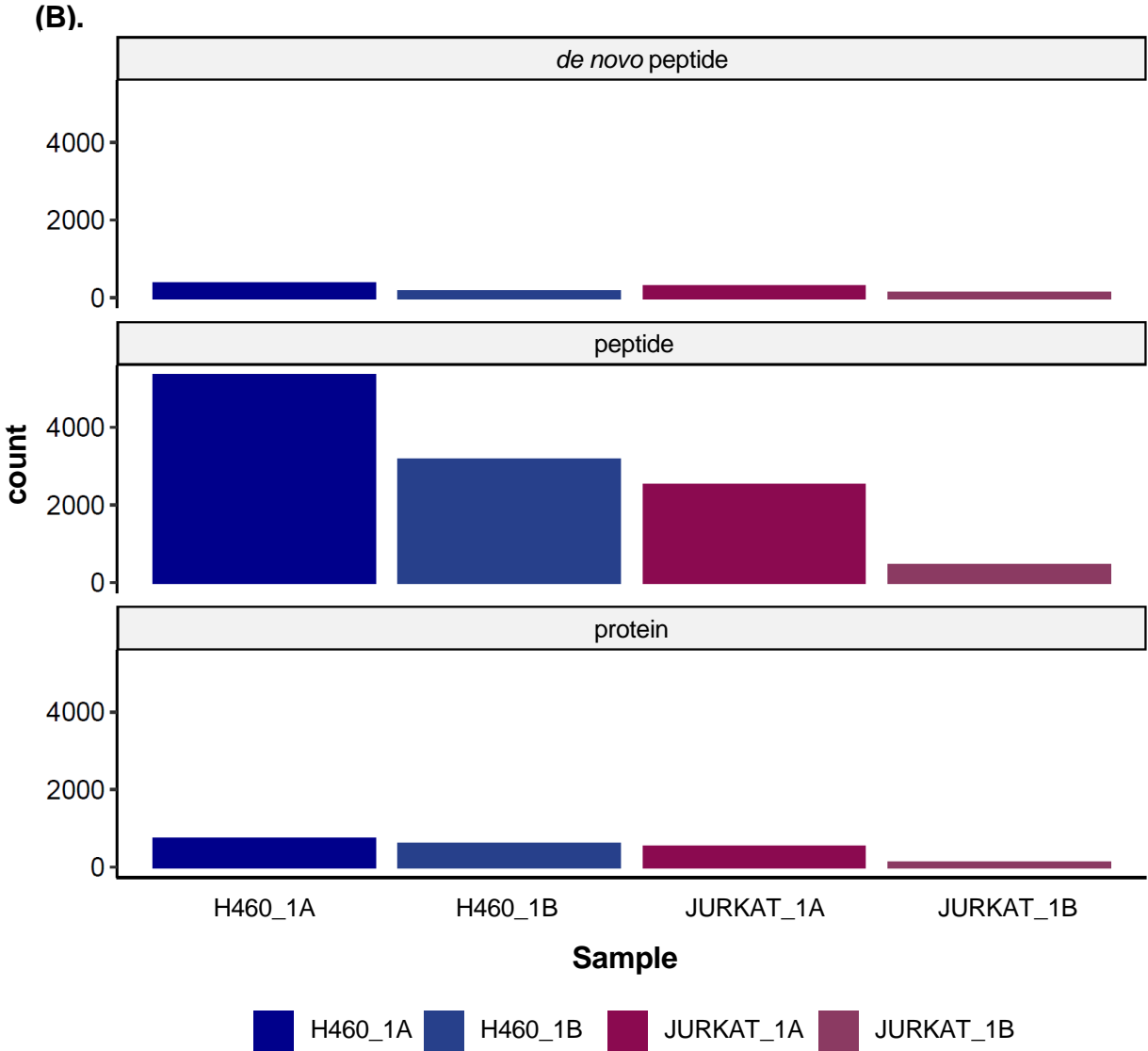

Supplement: Supplementary file 1 [file ijms-25-09564-s001.zip › Supplementary Figure S2.pdf]

Supplementary Figure S3

(A).

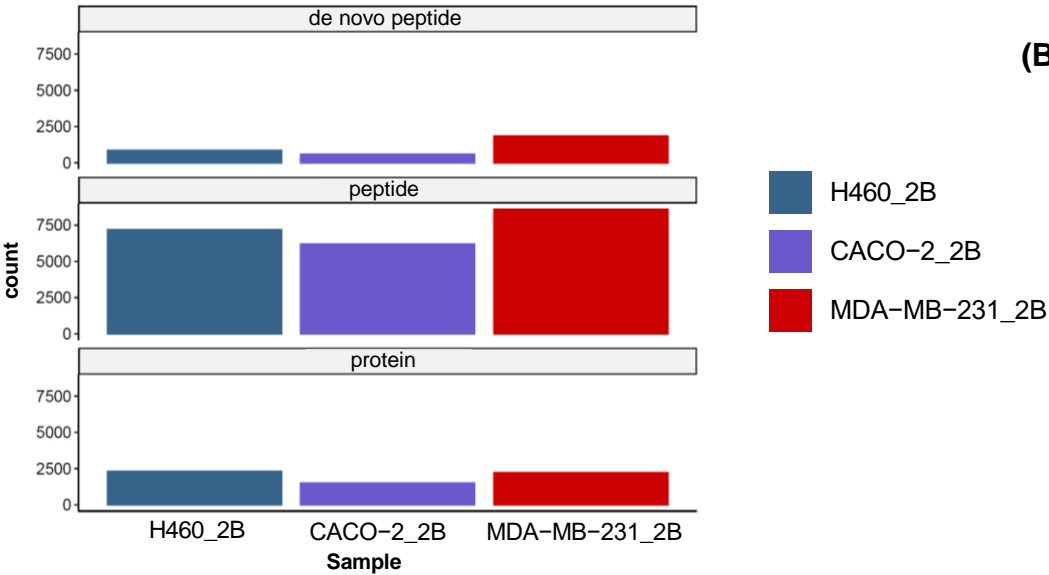

(B).

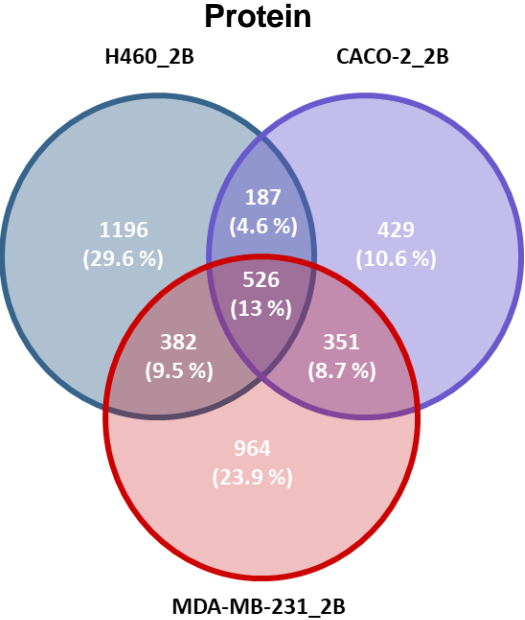

(C).

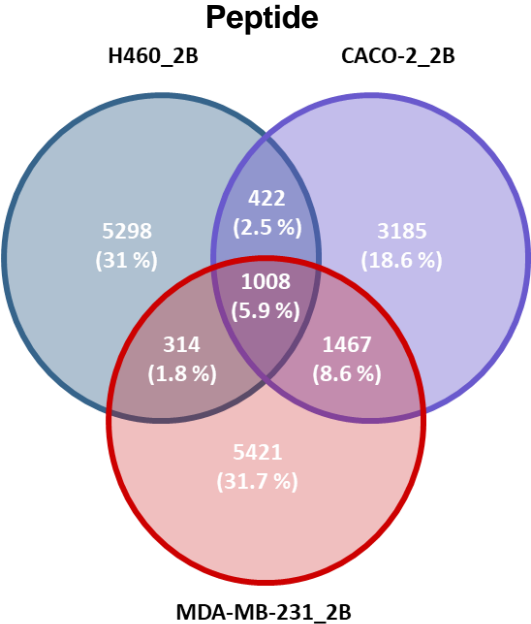

(D).

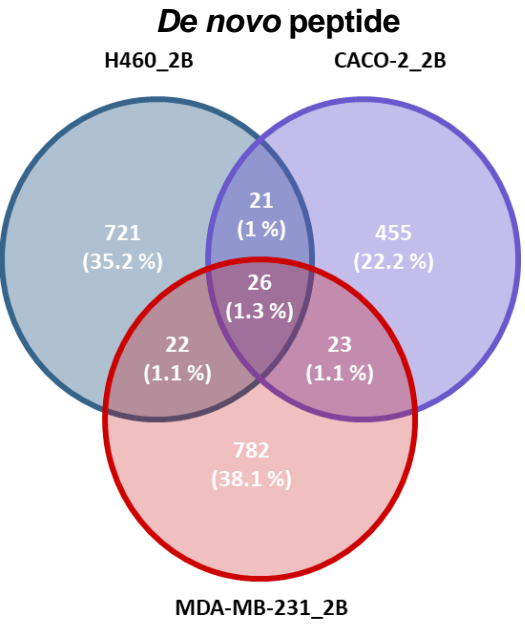

Supplement: Supplementary file 1 [file ijms-25-09564-s001.zip › Supplementary Figure S3.pdf]

Supplementary Figure S4

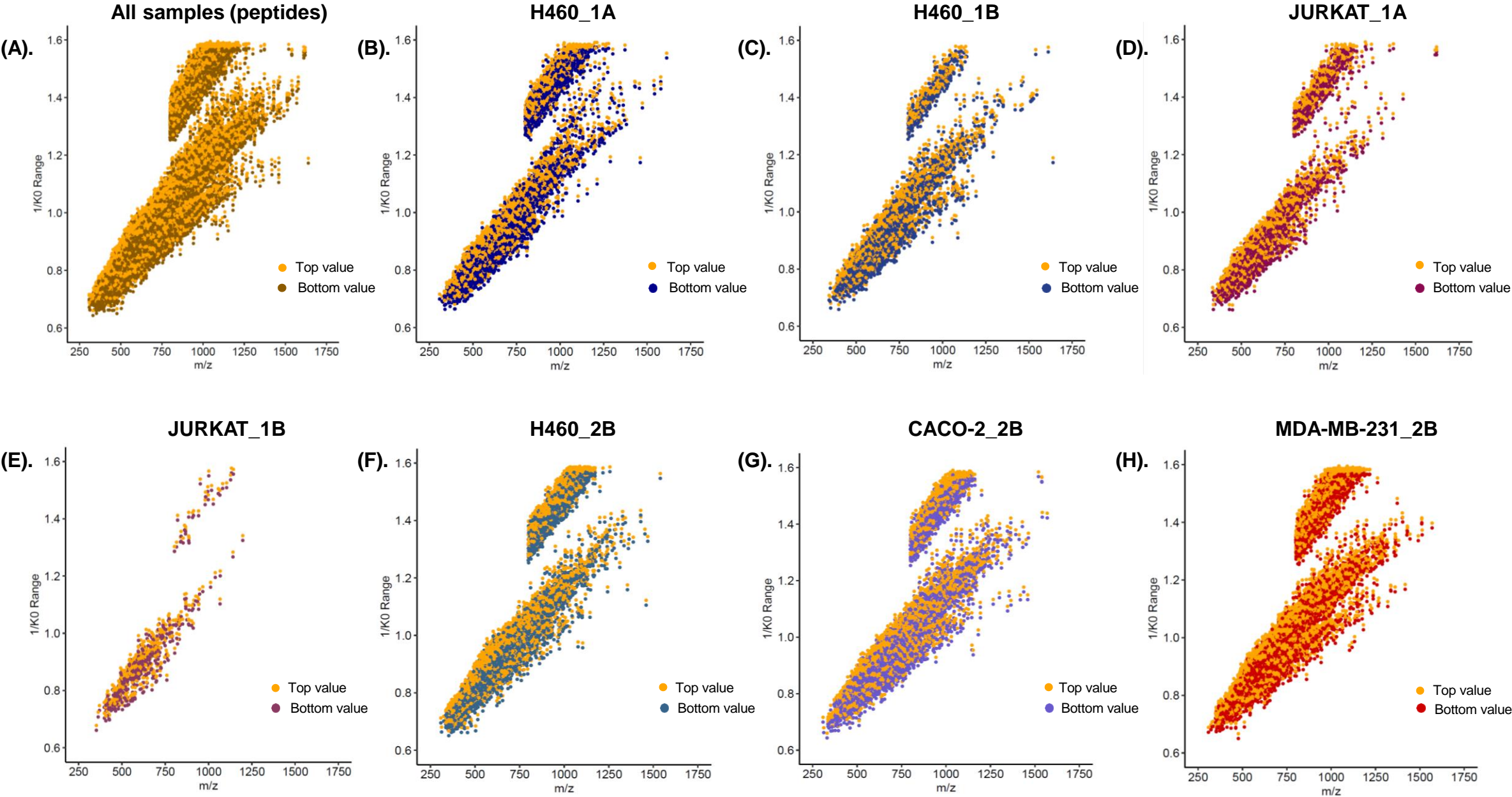

Supplement: Supplementary file 1 [file ijms-25-09564-s001.zip › Supplementary Figure S4.pdf]

Supplementary Figure S5

All samples (*de novo* peptides)

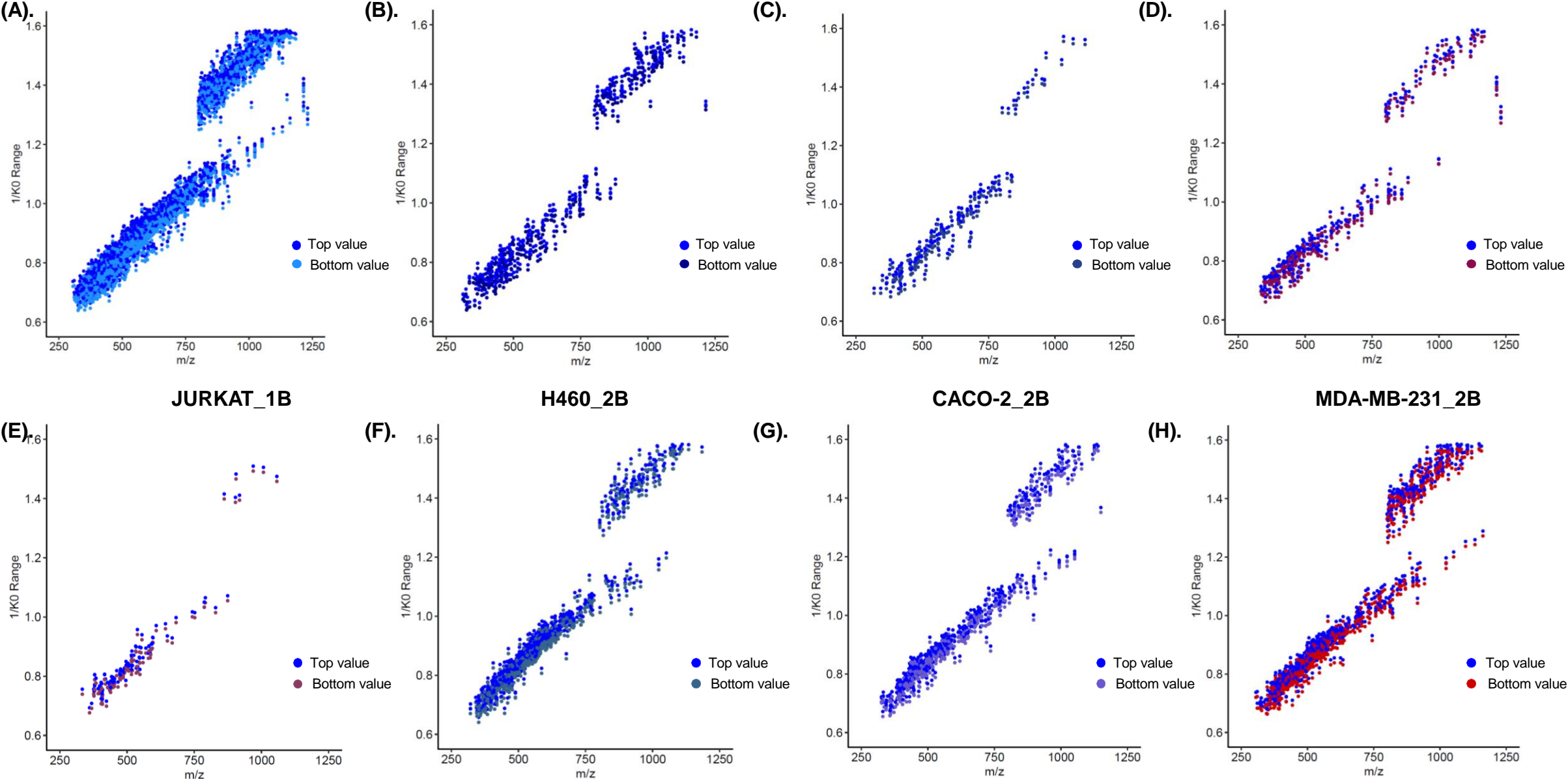

Supplement: Supplementary file 1 [file ijms-25-09564-s001.zip › Supplementary Figure S5.pdf]

Supplementary Figure S6.

(A).

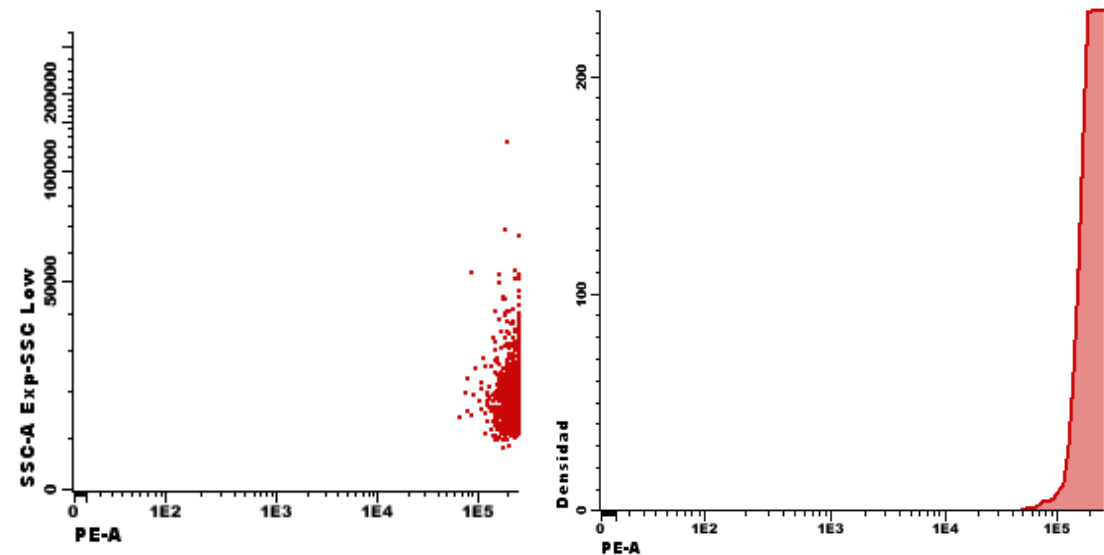

(B).

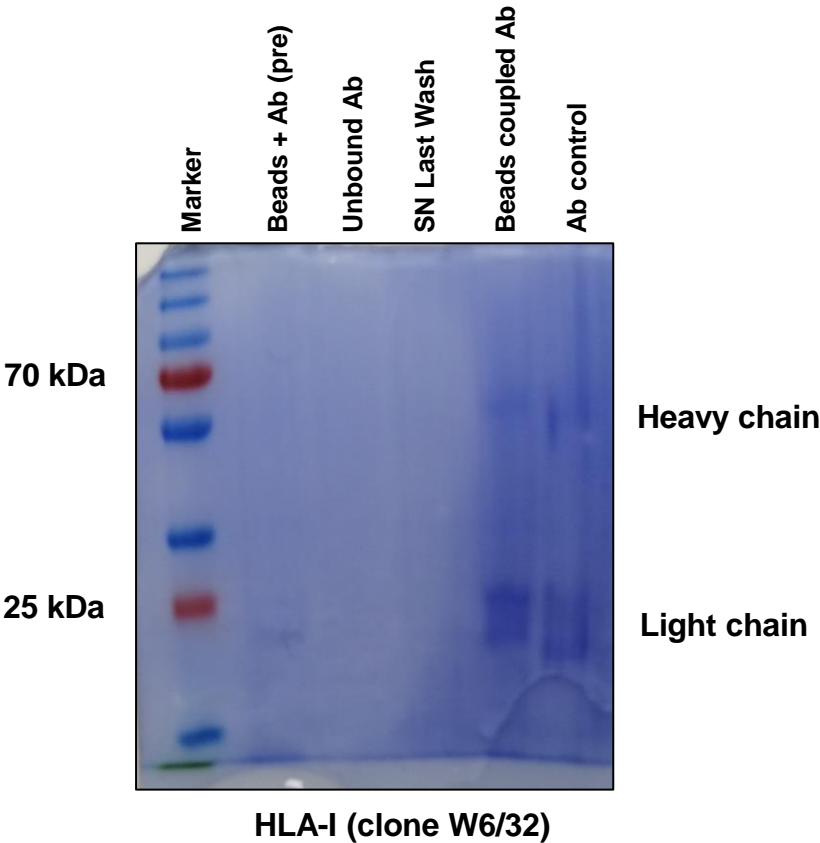

(C).

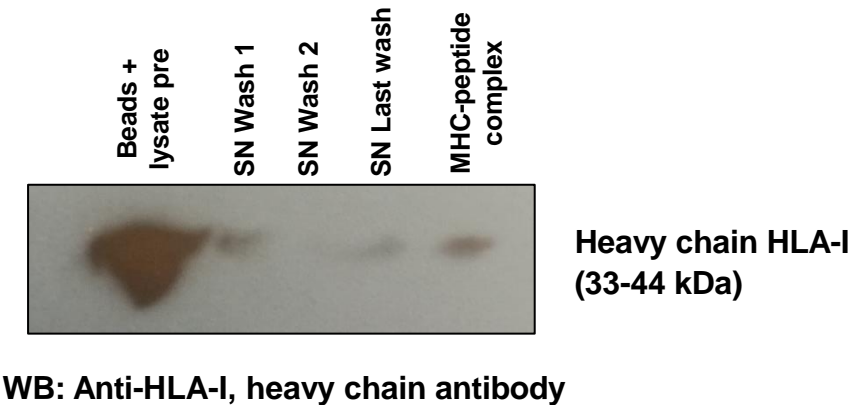

Supplement: Supplementary file 1 [file ijms-25-09564-s001.zip › Supplementary Figure S6.pdf]
